# High-Precision Spatial Interpolation of Meteorological Variables in Complex Terrain Using Machine Learning Methods

**DOI:** 10.3390/s26072167

**Published:** 2026-03-31

**Authors:** Shuangping Li, Bin Zhang, Bo Shi, Qingsong Ai, Yuxi Zeng, Xuanyao Yan, Hao Chen, Huawei Wang

**Affiliations:** 1 Changjiang Spatial Information Technology Engineering Co., Ltd., Wuhan 430010, China; 2 State Key Laboratory of Precision Geodesy, Innovation Academy for Precision Measurement Science and Technology, CAS, Wuhan 430077, China; 3 Institute of Climate Application Research/CPRM/CIC-FEMD/KLME/ILCEC, Nanjing University of Information Science and Technology, Nanjing 210044, China

**Keywords:** machine learning, spatial interpolation, complex terrain, eXtreme gradient boosting

## Abstract

This study has explored the effectiveness of machine learning methods for high-precision spatial interpolation of meteorological variables, aiming to provide accurate atmospheric delay corrections for high-precision edge and corner nets observation in complex-terrain environments such as the Xiluodu Hydropower Station, thereby enhancing the accuracy of deformation monitoring. Considering the significant limitations of traditional interpolation methods such as Inverse Distance Weighting (IDW) and Ordinary Kriging (OK) in capturing spatial variability under complex topographic conditions, we systematically introduced machine learning algorithms including Random Forest (RF)and eXtreme Gradient Boosting (XGBoost, XGB) to compare their performance with traditional methods for high-density interpolation of sparsely distributed temperature, relative humidity, and surface pressure, respectively. Concurrently, we proposed an enhanced XGB model incorporating center-point features (XGB-C) which frames spatial interpolation as a supervised learning problem that learns physical mapping from synoptic backgrounds to local microclimates instead of relying on geometric distances alone. The interpolation performance indices (RMSE, MAE, and R^2^) were evaluated with daily meteorological observations from 47 stations (38 for training, 9 for testing) during 2023–2024. Results demonstrate that machine learning methods significantly outperform traditional approaches, with XGB-C achieving the highest accuracy (R^2^ ≈ 1.00 for pressure, 0.97 for humidity, 0.83 for temperature). Moreover, the interpolation performance also exhibits a dependence on seasons and the station location. Greater challenges are shown in the summer season and in the “Urban and Built-Up” and “Croplands” areas. These findings highlight the substantial advantages of machine learning, particularly the proposed XGB-C, for meteorological interpolation in mountainous hydropower station environments where accurate atmospheric correction is crucial for deformation monitoring. This also lays a solid foundation for developing operational ML-based interpolation models trained with high-quality labels derived from unmanned aerial vehicle (UAV) remote sensing data.

## 1. Introduction

In edge-angle network observations, the measurements are highly susceptible to atmospheric refraction effects and local meteorological conditions, resulting in bending of the propagation path and thereby creating discrepancies between the observed and the actual distances [1,2]. Particularly in hydropower station areas which are typically located in mountainous valleys and large water body environments, the characteristic unstable fluctuations in meteorological elements such as temperature, humidity, and atmospheric pressure further exacerbate uncertainties in atmospheric refraction. The unstable fluctuations include topography, windward or leeward slopes, horizontal distance from water surfaces, elevation gradients, slope angles, and aspect orientations significantly that exhibit complex local circulation patterns, such as valley-mountain wind systems [3,4,5,6]. In addition, the meteorological stations are sparsely and unevenly distributed, which can only provide local, discrete, limited spatial data, resulting in very limited available meteorological data over the whole region [7,8]. Thus, spatial interpolation with sparse observations is usually implemented. However, achieving high-precision spatial interpolation of meteorological variables remains extremely challenging [7,9,10], especially complex terrain. Traditional interpolation methods such as Inverse Distance Weighting (IDW) and the Kriging model exhibit limitations in characterizing spatial variability when confronted with complex topography and nonlinear meteorological processes [11,12,13,14].

In recent decades, machine learning techniques are widely used to address meteorological issues, and deep learning methods have emerged as powerful tools for downscaling [15,16,17,18,19,20,21]. There is a significant surge in the application of machine learning and deep learning methods for spatiotemporal interpolation of meteorological observations [22,23,24]. Due to the inherent advantages in capturing nonlinear relationships, integrating multi-source data, and automatic feature selection, numerous studies have combined machine learning with traditional interpolation methods for high-precision modeling of meteorological elements such as temperature and precipitation [25,26,27]. Appelhans et al. comprehensively assessed the interpolation performance of monthly mean temperature over Mount Kilimanjaro, Tanzania with 14 machine learning methods. The results found that regression tree-based algorithms generally outperformed traditional methods like cokriging in spatial prediction tasks [28], and the machine learning algorithms demonstrate clear advantages in quantitative metrics such as RMSE and MAE [29,30]. Regarding the machine learning algorithms, a notable example involves the application of XGB, Support Vector Regression (SVR), and multiple linear regression models for temperature downscaling in mountainous regions using ERA5 land reanalysis data [31]. These approaches significantly improve both the spatial resolution and accuracy of ERA5-land’s 9 km resolution temperature data, demonstrating the substantial potential of machine learning models in downscaling applications [32,33]. In addition, RF has proven to be one of the most successful machine learning algorithms for spatial interpolation of meteorological variables [34,35]. Hengl et al. proposed RF as a generic framework for predictive modeling of spatial and spatiotemporal variables, demonstrating its effectiveness across diverse environmental datasets [36]. The ranger implementation by Wright and Ziegler has enabled efficient processing of high-dimensional meteorological data [37], while Sekulić et al. specifically developed RF spatial interpolation methods that outperform traditional geostatistical approaches [38]. In addition, some studies attribute the advantage of the machine learning method to its ability to capture the relationships among different features [39]. For meteorologically sparse and mountainous areas, such as the Tibetan Plateau, former studies have begun applying ensemble machine learning methods for regional-scale temperature interpolation [40,41]. Results indicate that Random Forest, Support Vector Machines, and Gaussian Process Regression all outperform traditional methods, particularly demonstrating greater robustness in data-sparse regions [42]. However, insufficient attention has been paid to the efficiency of machine learning methods in the interpolation of meteorological variables of hydropower stations, where the terrain is complex and the observation is sparse.

This study aims to investigate whether the machine learning methods are effective for interpolating meteorological fields (the process of creating continuous spatial distributions of meteorological variables over the entire study area based on discrete point observations from stations) at the Xiluodu Hydropower station in China. Therefore, meteorological corrections along any survey line within the region can be obtained through interpolation from meteorological field information, thus further enhancing the accuracy of electromagnetic distance measurement and providing reliable technical support for deformation monitoring. To address the challenges posed by complex-terrain mountainous valleys and large water bodies, we propose XGB-C, an enhanced eXtreme Gradient Boosting model that integrates center-point features. Unlike conventional approaches relying solely on geometric distances, XGB-C reformulates spatial interpolation as a supervised learning task, learning the physical mapping from a representative synoptic background to local microclimates. By explicitly incorporating reference-anchored features, the model captures complex nonlinear interactions between topography and meteorological states, improving both interpolation accuracy and interpretability. By systematically comparing XGB-C against a suite of benchmark methods, ranging from traditional geostatistical techniques like Ordinary Kriging (OK) and Inverse Distance Weighting (IDW) to advanced machine learning algorithms such as Random Forest (RF) and standard XGB, we explore the optimal high-precision interpolation approaches for different meteorological variables (temperature, humidity, and surface pressure) in heterogeneous environments. This approach provides a physically informed framework for high-resolution meteorological predictions.

Regarding the selection of variables, we focus on temperature, humidity, and surface pressure because these are the only meteorological variables available from the observation stations in our study area, the three elements affect accuracy is through their modulation of the atmospheric refractive index which are the mainly influence for electronic distance measurement (EDM) as in high-precision edge and corner network observations. This modulation alters the propagation velocity of electromagnetic waves, generating a systematic scale error that is directly proportional to the measured distance. As the most significant variable, temperature directly governs atmospheric density. An increase in temperature lowers air density, which in turn reduces the refractive index and accelerates wave propagation. Conversely, atmospheric pressure serves as a direct indicator of density: higher pressure corresponds to denser air, leading to an increased refractive index and a consequent deceleration of the wave. Humidity, representing the concentration of water vapor molecules, also influences propagation velocity, particularly as vapor pressure rises. In practice, the impact of humidity is typically subordinate to that of temperature and pressure. However, in high-precision deformation monitoring or high-accuracy traversing or under extreme conditions of high temperature and relative humidity, the contribution of humidity to the overall refractive error becomes significant and must be incorporated into rigorous meteorological corrections. Quantitatively, the error induced by temperature is approximately 2 mm per kilometer of distance for every 1 °C deviation. Meanwhile, 1 mmHg deviation in atmospheric pressure yields a distance error of approximately 1.3 mm per kilometer. Collectively, these meteorological errors, if unmitigated, can substantially degrade the reliability of precision measurement and deformation monitoring outcomes. The remainder of this paper is organized as follows: Section 2 describes data and interpolation methods; Section 3 introduces the characteristics of observed meteorological variables; Section 4 presents the comparison of interpolation accuracy using the conventional interpolation and machine learning methods; Section 5 summarizes and discusses the findings.

## 2. Data and Methods

### 2.1. Data Sources

**Figure 1:**
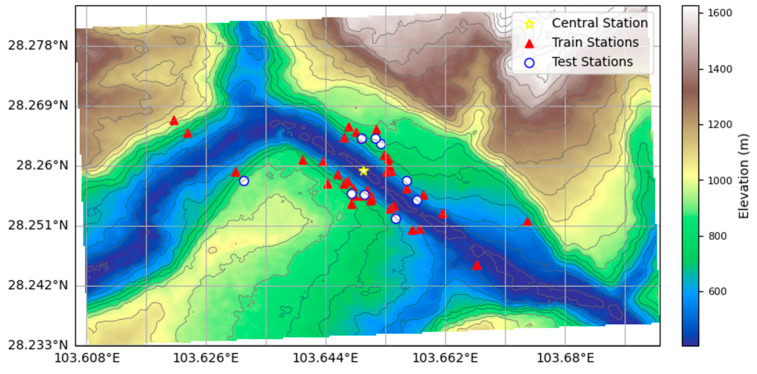
Distribution of meteorological (38 train stations with red triangles, and 9 test stations with white dots) and topographic height in Xiluodu hydropower station. The star-marked station (QTP15-1) is the central station.

**Figure 2:**
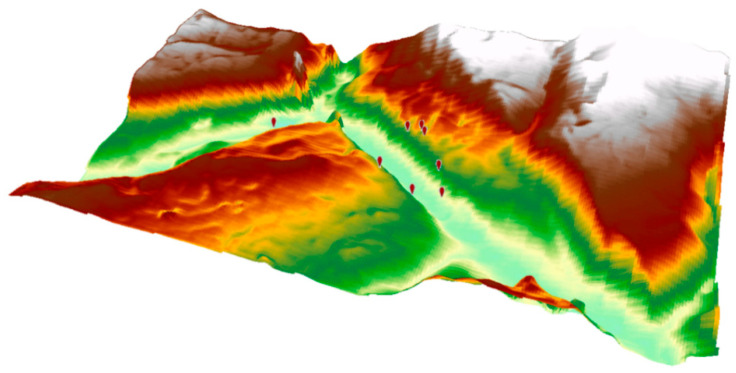
3D terrain visualization in the Xiluodu dam hub area. The surface colors represent elevation, where light green/yellow indicates low-lying valley floors and dark brown/white represents higher mountain ridges and peaks, and the red symbols denote the stations.

The dataset in this study consists of daily meteorological observations from 47 stations located in the hydropower dam area, covering the period from 1 January 2023 to 31 December 2024, and the raw observations were initially recorded at 20 min intervals. During the preprocessing stage, strong physical constraints were applied to ensure consistency with atmospheric principles, including removing any values outside physically plausible ranges (e.g., 0–100% for relative humidity). To avoid introducing additional noise, missing observations were not artificially imputed. Instead, we performed temporal sampling by aggregating the original high-frequency observations into daily mean values. For model training and evaluation, a station-based random spatial sampling strategy was implemented. The 47 stations were partitioned into spatially mutually exclusive sets: a training set (38 stations) and an independent testing set (9 stations). While the training stations cover the entire dam site, the testing stations are strategically distributed across the region to ensure relatively uniform coverage for independent accuracy assessment The spatial distribution of training and testing stations within the study area is illustrated in Figure 1, and the 3D terrain visualization is presented in Figure 2. Detailed geographic information of the test stations, including the longitude, latitude, terrain height, slope, aspect type and land-use type, is listed in Table 1. Table 1 summarizes the characteristics of the test stations, showing the complex terrain of the study area. Elevations range from 472.47 m (QHV02-XZ) to 892.29 m (QVDR11), with slopes spanning 14.91° to 52.06° at the steepest site (QVDR08-2). The stations occupy diverse land-use types, including Urban and Built-Up areas, Croplands, Evergreen Broadleaf Forests, and Woody Savannas, and are strategically distributed across different sectors (South-West, South, North-West, and North-East), capturing the spatial heterogeneity of topography and land cover.

The digital elevation model (DEM) used in this study was derived from the Shuttle Radar Topography Mission (SRTM) dataset, acquired by the Endeavour Space Shuttle, with a spatial resolution of 30 m. The data were obtained from the Geospatial Data Cloud of the Chinese Academy of Sciences (https://www.gscloud.cn/home).

### 2.2. Interpolation Methods

#### 2.2.1. Inverse Distance Weighting (IDW)

IDW is one of the most widely used deterministic interpolation methods in geospatial studies, and often serves as a baseline approach [43,44]. As a representative member of distance-weighting methods, IDW is based on Tobler’s first law of geography, which assumes that nearby locations are more similar than those farther apart. The weight assigned to each observation is inversely proportional to its distance, giving greater influence to nearby samples and smaller influence to more distant ones. The interpolated value is obtained by computing a weighted average of neighboring observations.

Given n is the known sample points $\left(x_{i},y_{i}\right)$, whose observed values are $z_{i}\left(i=1,2,\ldots ,n\right)$, the interpolation $\hat{z}\left(x_{0},y_{0}\right)$ at the predicted position $\left(x_{0},y_{0}\right)$ is defined as a weighted average form:

(1) $$\hat{z}\left(x_{0},y_{0}\right)=\frac{\sum_{i=1}^{n}w_{i}z_{i}}{\sum_{i=1}^{n}w_{i}}$$

The weight $w_{i}$ is related to the Euclidean distance between the interpolated point and the sample point, and is usually defined as:

(2) $$w_{i}=\frac{1}{{\left[\sqrt{{\left(x_{0}-x_{i}\right)}^{2}+{\left(y_{0}-y_{i}\right)}^{2}}\right]}^{p}}$$

#### 2.2.2. Ordinary Kriging (OK)

OK is a geostatistical interpolation technique that relies on variogram modeling and structural analysis to provide the best linear unbiased estimator for unsampled locations [39]. Among its variants, Ordinary Kriging (OK) is one of the most frequently applied methods, and is commonly employed as a benchmark in spatial prediction studies [44]. For this reason, OK is also selected in this study for accuracy comparison.

For the target point $s_{0}$, the OK estimator is

(3) $$\hat{Z}\left(s_{0}\right)=\sum_{i=1}^{n}\lambda_{i}Z\left(s_{i}\right)$$

where $\lambda_{i}$ is the weight assigned to the sample point, which must satisfy the unbiasedness constraint:

(4) $$\sum_{i=1}^{n}\lambda_{i}=1$$

The weight $\lambda_{i}$ is determined by the following Kriging equation:

(5) $$\left\{\begin{array}{l} \sum_{j=1}^{n}\lambda_{j}\gamma \left(s_{i}-s_{j}\right)+\mu^{\prime }=\gamma \left(s_{i}-s_{0}\right),i=1,2,\ldots ,n\\ \sum_{j=1}^{n}\lambda_{j}=1 \end{array}\right.$$

Here, $\mu^{\prime }$ is the Lagrange multiplier introduced to enforce the unbiasedness constraint, and γ represents the spatial autocorrelation defined by the semivariogram function:

(6) $$\gamma \left(h\right)=\frac{1}{2N\left(h\right)}\sum_{i=1}^{N\left(h\right)}{\left[Z\left(s_{i}\right)-Z\left(s_{i}+h\right)\right]}^{2}$$

The semivariogram $\gamma \left(h\right)$ is defined as the average squared difference between sample pairs separated by a distance interval h, where $N\left(h\right)$ denotes the number of sample pairs within the distance interval. After determining the weights, they are substituted into the interpolation equation to obtain the optimal interpolated value.

#### 2.2.3. Random Forest (RF)

RF proposed by Breiman [29], is an ensemble learning algorithm that constructs a collection of decision trees as base learners. By employing the principle of bagging (bootstrap aggregating), RF aggregates multiple decision trees to improve interpolation accuracy. In contrast to conventional decision trees, RF introduces randomness in both sample and feature selection. Specifically, each tree is trained using a bootstrap sample drawn from the original dataset, while a random subset of input features (e.g., elevation, longitude, latitude) is selected at each node to determine the optimal split. This procedure results in a diverse ensemble of decision trees with reduced correlation. Model performance is internally assessed using out-of-bag samples that are not included in the bootstrap datasets. For regression-based interpolation, the final interpolated value is obtained by averaging the outputs of all individual trees [45,46]. This randomization strategy enhances model generalization, mitigates overfitting, and improves robustness against noise. Owing to these advantages, RF has been widely applied in regression-based spatial interpolation studies. A detailed description of the algorithm can be found in [29].

The training sample D can be expressed as:

(7) $$D={\left\{\left(x_{i},y_{i}\right)\right\}}_{i=1}^{N}$$

where $x_{i}$ represents the feature vectors of the i th sample (e.g., longitude, latitude, elevation), and $y_{i}$ is the observed value (such as temperature or precipitation).

Random Forest integrates B independent decision tree models $h_{b}\left(x\right)$ to obtain the prediction:

(8) $${\hat{f}}_{\left(x\right)}=\frac{1}{B}\sum_{b=1}^{B}h_{b}\left(x\right)$$

Each tree $h_{b}$ is trained on bootstrap samples and is partitioned using only random subset features at each node.

#### 2.2.4. eXtreme Gradient Boosting (XGB)

The eXtreme Gradient Boosting (XGB) is a tree-based ensemble learning algorithm that builds an additive model by sequentially fitting regression trees to minimize a specified loss function with regularization [30,47]. Owing to its ability to handle nonlinear relationships, feature interactions, and collinearity, XGB has been widely applied in regression-based spatial interpolation problems.

Given a training dataset expressed as Equation (7), the XGB model represents the interpolated value as the sum of K regression trees:

(9) $${\hat{y}}_{i}=\sum_{k=1}^{K}f_{k}\left(x_{i}\right),f_{k}\in F$$

where F represents the function space of the regression tree.

The overall optimization objective of the model is defined as:

(10) $$\Gamma =\sum_{i=1}^{N}l\left(y_{i},{\hat{y}}_{i}\right)+\sum_{k=1}^{K}\Omega \left(f_{k}\right)$$

Among them, $l\left(y_{i},{\hat{y}}_{i}\right)$ is the loss function; $\Omega \left(f_{k}\right)$ is a regularization term used to control model complexity and prevent overfitting.

(11) $$\Omega \left(f_{k}\right)=\gamma T+\frac{1}{2}\lambda \sum_{j=1}^{T}{w_{j}}^{2}$$

where T is the number of leaf nodes in the tree, $w_{j}$ is the output weight of the jth leaf node, γ and λ are regularization parameters.

The tree model for each iteration is approximately optimized using a second-order expansion:

(12) $$L^{\left(t\right)}≃\sum_{i=1}^{N}\left[g_{i}f_{t}(x_{i})+\frac{1}{2}h_{t}f_{t}^{2}(x_{i})\right]+\Omega (f_{t})$$

where $g_{i}$ and $h_{i}$ denote the first- and second-order gradients, respectively.

#### 2.2.5. XGB with Center-Point Features (XGB-C)

To capture complex nonlinear interactions between topographic features and meteorological states, we developed an enhanced model termed XGB-C, based on eXtreme Gradient Boosting (XGB). The primary novelty of XGB-C lies in the construction of a reference-anchored feature space, which reformulates spatial interpolation as a supervised learning task. Instead of relying solely on geometric distance metrics, the model learns the physical mapping between a reference synoptic background and local microclimatic conditions.

**Figure 3:**
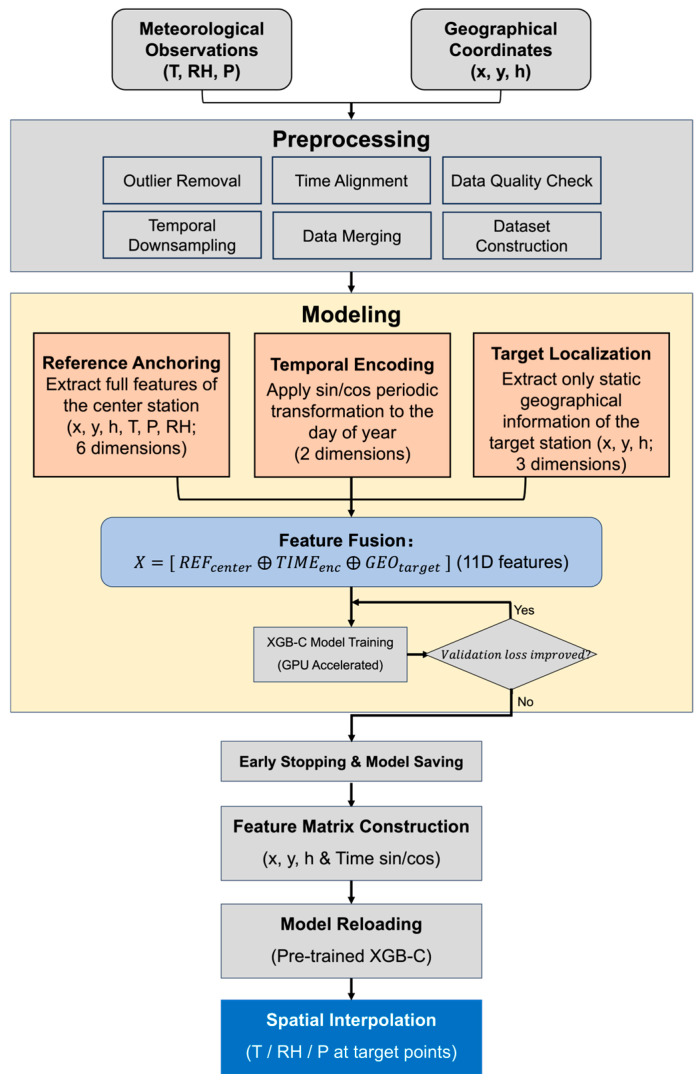
The overall framework of the proposed XGB-C modeling process. The symbol ⨁ denotes the vector concatenation operation.

As illustrated in Figure 3, the feature engineering strategy incorporates physical constraints by augmenting local geographic attributes with regional background states. Specifically, the modeling core constructs an 11-dimensional feature vector X, consisting of three components. The first component comprises the three-dimensional coordinates (longitude, latitude, elevation) of target stations. The second component includes the six-dimensional attributes of a designated reference station, combining its 3D coordinates with instantaneous meteorological observations (temperature, pressure, and humidity). The remaining two dimensions encode the Day of Year (DOY) using sine and cosine transformations to represent climatological seasonality while preserving temporal continuity. The inclusion of the reference (center) station’s full meteorological state serves as a proxy for the large-scale synoptic background, enabling the model to leverage multivariate physical coupling to disentangle regional climate trends from local orographic modifications. Crucially, during the training phase, the center station is strictly excluded from the target dataset. This constraint prevents the model from learning a trivial identity mapping and instead forces it to generalize the physical transfer function that governs how the background flow evolves across heterogeneous terrain.

Following a preprocessing phase characterized by rigorous outlier removal, time alignment, and temporal down sampling, the modeling core constructs an 11-dimensional feature fusion vector (Figure 3). The iterative learning process, accelerated by GPU and regulated by an early stopping mechanism, ensures that the resulting emulator is both computationally efficient and physically consistent for final spatial interpolation.

### 2.3. Accuracy Assessment

The interpolation performance was evaluated by four commonly adopted statistical metrics: Root Mean Square Error (RMSE), Mean Absolute Error (MAE), the coefficient of determination (R^2^), and the Anomaly Correlation Coefficient (ACC). RMSE measures the sensitivity of estimated values to sampling data and highlights the influence of extreme deviations [48], while MAE quantifies the average magnitude of prediction errors, providing a direct estimate of the possible error range [49]. In addition, ACC, a widely used skill metric in atmospheric sciences, was employed to assess the linear agreement between interpolated and observed spatial fields. ACC is defined as the anomaly correlation coefficient, representing the cosine of the angle between two anomaly vectors. Higher ACC values indicate stronger consistency in spatial patterns and improved interpolation skill. In summary, smaller RMSE and MAE values, together with larger R^2^ and ACC values, correspond to higher interpolation accuracy.

(13) $$RSME=\sqrt{\frac{1}{N}\sum_{i=1}^{N}{\left(x_{i}-{\hat{x}}_{i}\right)}^{2}}$$

(14) $$MAE=\frac{1}{N}\sum_{i=1}^{N}\left|x_{i}-{\hat{x}}_{i}\right|$$

(15) $$R^{2}=1-\frac{\sum_{i=1}^{N}{\left(x_{i}-{\hat{x}}_{i}\right)}^{2}}{\sum_{i=1}^{N}{\left(x_{i}-{\bar{x}}_{i}\right)}^{2}}$$

(16) $$ACC=\frac{\sum_{i=1}^{n}\left(x_{i}-\bar{x}\right)\left(y_{i}-\bar{y}\right)}{\sqrt{\sum_{i=1}^{n}{\left(x_{i}-\bar{x}\right)}^{2}}\sqrt{\sum_{i=1}^{n}{\left(y_{i}-\bar{y}\right)}^{2}}}$$

where n is the number of samples, ${\hat{x}}_{i}$ is the predicted value of the ith data point, which is the estimated value given by the model, $x_{i}$ is the true value of the ith data point, which is the actual observed target value. $\left|x_{i}-{\hat{x}}_{i}\right|$ is the absolute error of the ith predicted value, ${\bar{x}}_{i}$ is the average of the true value $x_{i}$.

## 3. Characteristics of Meteorological Variables

### 3.1. Temporal Variations

**Figure 4:**
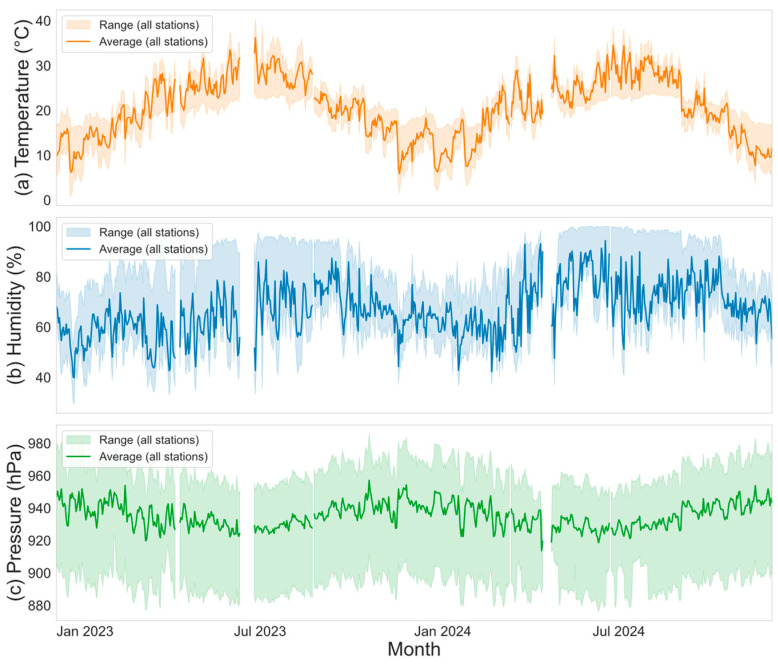
Temporal variations in daily (**a**) air temperature, (**b**) relative humidity and (**c**) surface pressure at all observation stations.

Understanding the temporal evolution of key meteorological variables is a prerequisite for identifying the dominant climatic patterns and assessing the consistency of observations across stations. Such analysis provides essential context for subsequent correlation and modeling studies, as it reveals whether the regional atmosphere is governed primarily by large-scale circulation or modulated by local topographic and environmental effects. Figure 4 illustrates the temporal variations of three key meteorological variables—temperature, relative humidity, and surface pressure—across multiple stations in the study area over approximately two years.

The temperature series (Figure 4a) exhibits pronounced seasonal variation, with higher values in summer (approximately June to August) and lower values in winter (approximately December to February), consistent with the typical annual cycle. While individual stations show moderate variability, the overall trend is largely coherent, indicating that regional climate is primarily influenced by large-scale atmospheric circulation, with local factors contributing to station-specific deviations. The black curve representing the mean temperature across all stations provides a clearer depiction of the overall seasonal pattern, smoothing out station-specific fluctuations while retaining the characteristic annual cycle.

The relative humidity series (Figure 4b) is more variable and does not display as distinct an annual cycle as temperature. However, a general seasonal pattern is observed, that is, humidity is higher during summer and lower in winter. The fluctuations are more pronounced. Substantial inter-station differences suggest that humidity is strongly affected by local factors such as topography and water bodies, leading to greater spatial heterogeneity compared to temperature. The mean humidity curve captures the overall trend but still reflects noticeable variability due to the wide dispersion among stations.

The surface pressure (Figure 4c) exhibits frequent short-term fluctuations superimposed on longer-term trends. Variations among stations highlight the spatial heterogeneity of the pressure field, influenced by synoptic-scale weather systems such as cyclones and anticyclones. The mean pressure curve smooths short-term oscillations, providing a more representative view of the temporal evolution of surface pressure across the study area.

### 3.2. Spatial Deviation

**Figure 5:**
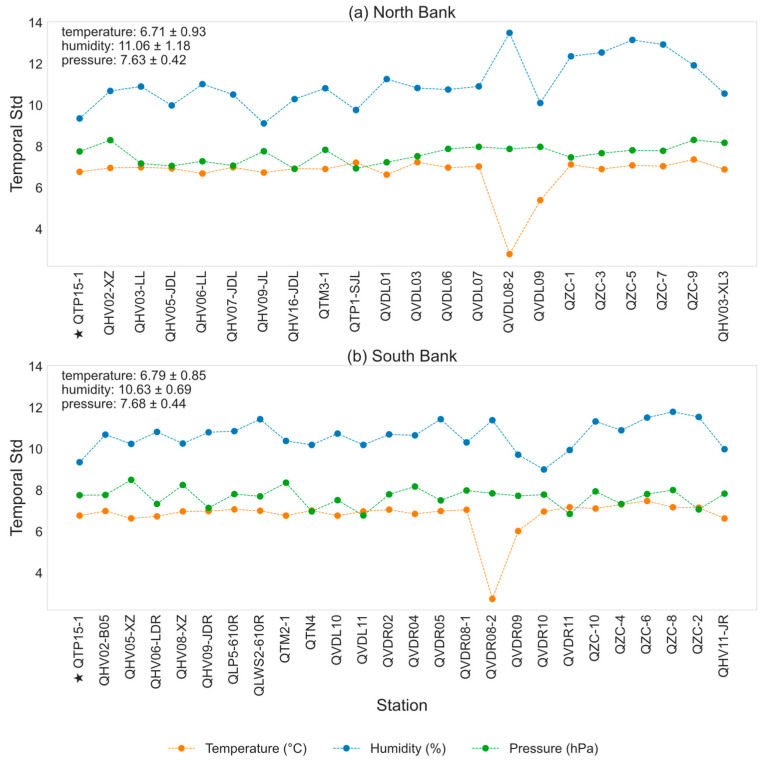
Temporal standard deviation of air temperature (orange dotted lines), relative humidity (blue dotted lines), and surface pressure (green dotted lines) of observation stations on (**a**) north bank and (**b**) south bank. The star-marked station (QTP15-1) is the central station.

Quantifying the temporal variability of meteorological variables at individual stations provides an effective way to diagnose spatial differences in observational stability across the study area. Such differences reflect how local environmental factors, including topography and surface characteristics, modulate the temporal response of meteorological conditions to large-scale atmospheric forcing. This analysis therefore offers a physically meaningful measure of spatial deviation in temporal behavior, helping to evaluate the representativeness of station observations and the dynamic range of inputs for subsequent spatial interpolation. Figure 5 presents the temporal standard deviation (Std) of temperature, relative humidity, and surface pressure for each station over the two-year period. The Std quantifies the magnitude of temporal fluctuations at individual stations, and contrasts among stations directly indicate spatial heterogeneity in temporal stability within the study area.

Overall, the temporal variability of surface pressure (green dotted lines) remains relatively stable across most stations, implying relatively uniform pressure conditions across stations. Stations located on the south and north banks exhibit similar mean levels of temperature, relative humidity, and surface pressure, with the north bank showing slightly higher relative humidity compared to the south bank. At the north bank, station QVDL08-2 exhibits a relatively low temporal standard deviation of temperature, reflecting a flattened annual temperature cycle. Similarly, the south bank station QVDR08-2 shows a low temperature temporal Std.

**Figure 6:**
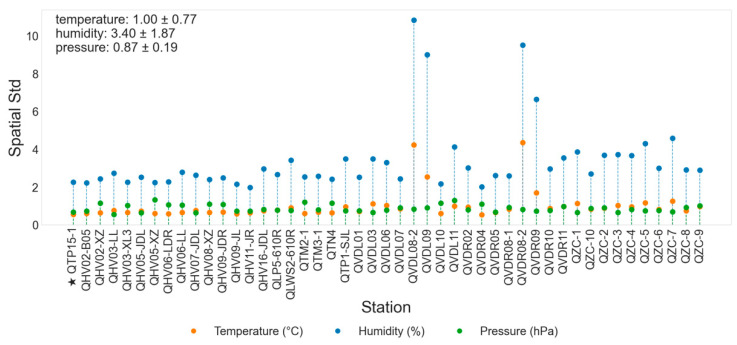
Spatial deviation of air temperature, relative humidity, and surface pressure across all observation stations. The star-marked station (QTP15-1) is the central station.

Figure 6 shows the temporal standard deviation of the spatial deviations, defined as the deviation of each station from the regional mean at each time step. As illustrated in Figure 6, both QVDR08-2 and QVDL08-2 display relatively high spatial Std in temperature and relative humidity, indicating pronounced deviations from the regional pattern compared to other stations. The distinctive meteorological characteristics observed at station QVDR08-2 can be largely attributed to its geographical setting and surface conditions. As shown in Table 1, this station is situated on the southern bank of the river, within an urban and built-up area, and is characterized by a moderate slope (approximately 52°) facing northeast. Its proximity to the river and surrounding urban surfaces exerts a significant influence on local thermal and moisture conditions. The presence of the water body enhances local humidity through evaporation and turbulent moisture exchange, which may be the reason why higher relative humidity is observed at QVDR08-2 compared with the regional average. Meanwhile, the urban land surface, with higher heat capacity and anthropogenic heat release, tends to moderate the diurnal and seasonal temperature variations. This results in lower temperatures in summer due to evaporative cooling and slightly higher winter temperatures due to heat storage and reduced nocturnal cooling, leading to a flattened annual temperature cycle.

Furthermore, the northeast-facing slope limits direct solar radiation during the daytime, further contributing to relatively lower daytime temperatures in summer. These combined topographic, hydrological, and urban effects create a distinctive microclimate at QVDR08-2, making its temporal and spatial variability patterns diverge from those of surrounding stations. Such findings underscore the necessity of considering terrain orientation, land use, and surface heterogeneity when analyzing local climate variability and in the development of AI-based spatial interpolation or predictive models.

## 4. Interpolation Analysis

### 4.1. Comparison of Interpolation Accuracy with Different Methods

**Figure 7:**
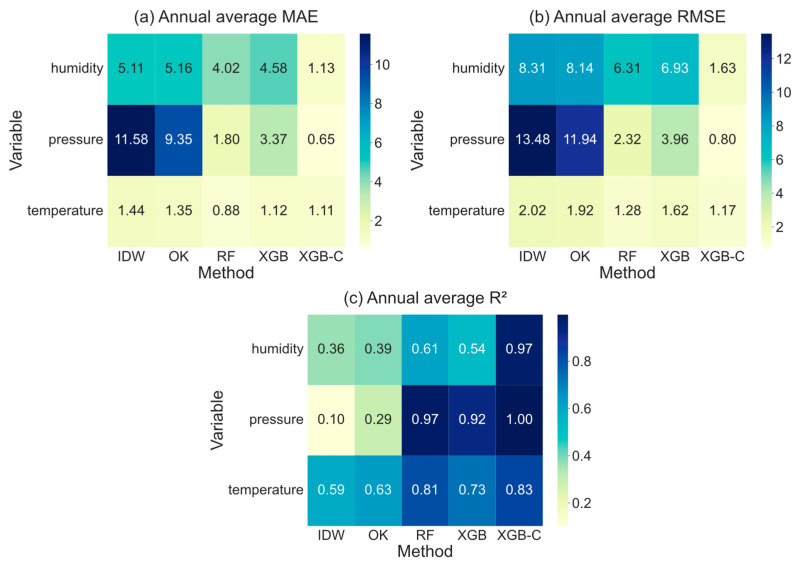
Interpolation accuracy heatmap. Annual average of (**a**) MAE, (**b**) RMSE and (**c**) R2 between the observed and interpolated air temperature, surface pressure and relative humidity using IDW, OK, RF, XGB and XGB-C methods.

To obtain high-precision meteorological data for the Xiluodu Hydropower Station hub area, we conducted tests with traditional methods (IDW and OK) and machine learning methods (RF, XGB and the proposed XGB-C model) in Section 2.2, and the accuracy statistics shown in Figure 7. As shown from the statistics, the performance of IDW and OK performed substantially worse than machine learning approaches. For all three meteorological variables (surface pressure, humidity, and temperature), both RMSE (Figure 7b) and MAE (Figure 7a) remained high, while the R^2^ (Figure 7c) is generally low. The MAE and RMSE of IDW for pressure reach 11.58 and 13.48, respectively, and those of OK were 9.35 and 11.94, with R^2^ values of only 0.10 and 0.29. These results highlight the limited capability of traditional interpolation methods to reconstruct spatial patterns under complex-terrain conditions, resulting in poor overall accuracy.

By contrast, machine learning-based interpolation methods (RF, XGB, and XGB-C) exhibit markedly superior performance, with substantially reduced MAE and RMSE and significantly improved R^2^. Among them, XGB-C consistently achieves the best accuracy across almost all variables. These findings indicate that machine learning methods have a higher ability to capture the nonlinear spatiotemporal variability of meteorological variables, and that the point-feature-enhanced XGB-C model yields the highest precision. Here, the central station for XGB-C is QTP15-1, of which geographical position balances the microclimatic influences of the north and south banks and plays a key role in site-specific monitoring. It is worth noting that we performed an exhaustive sensitivity analysis by rotationally designating each training station as the central station. While the results shown in Appendix A) were consistently similar, the current central station was selected for its unique spatial representativeness. Geographically, this station is situated on the river dam, effectively at the geometric center of the study area, which is shown in Figure 1. Given the distinct microclimatic differences between the north and south banks (due to aspect and slope), this central location allows it to serve as a balanced reference point that captures the background meteorology of the entire valley better than any station located solely on one bank.

A variable-wise comparison further confirmed these trends. For pressure, traditional methods result in extremely high errors and very low R^2^, whereas XGB-C achieved the highest accuracy (MAE = 0.65, RMSE = 0.80, R^2^ = 1.00). For humidity, IDW and OK produce large errors (MAE > 5.0, RMSE > 8.0) and low R^2^ (<0.40), while XGB-C shows remarkable improvement (MAE = 1.13, RMSE = 1.63, R^2^ = 0.97). For temperature, IDW and OK again show limited accuracy (R^2^ ≈ 0.6); RF achieves relatively good results (MAE = 0.88, RMSE = 1.28), but XGB-C provides the best overall performance with the highest R^2^ (0.83).

Within the machine learning methods, XGB-C consistently outperforms both RF and XGB, yielding the lowest MAE and RMSE and the highest R^2^ values (near 1). This demonstrates that integrating point-feature optimization into XGBoost can substantially enhance spatiotemporal interpolation performance.

### 4.2. Temporal Variation

**Figure 8:**
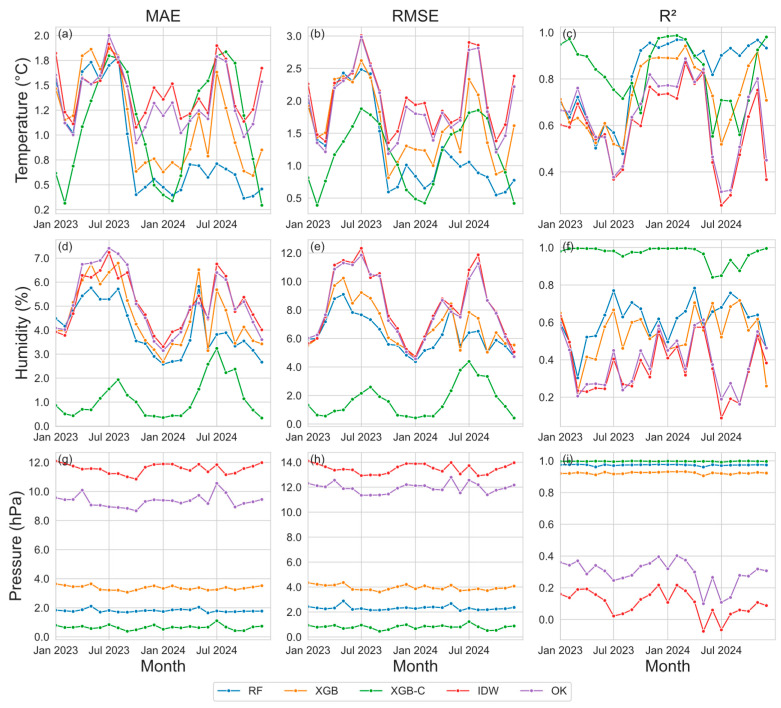
Monthly variation curves of MAE (the **left panel** (**a**,**d**,**g**)), RMSE (the **middle panel** (**b**,**e**,**h**)) and R2 (the **right panel** (**c**,**f**,**i**)) between the observed and interpolated air temperature, surface pressure and relative humidity using IDW, OK, RF, XGB and XGB-C methods.

Overall, the meteorological variables, especially the air temperature, exhibit an annual cycle (recall Figure 4). We further compare the interpolation performance indifferent calendar months (Figure 8). For temperature interpolation (Figure 8a–c), traditional methods (IDW and OK) show peak MAE (up to 1.5–2.0 °C) and RMSE (up to 2.0–2.8 °C) values in summer that decrease in winter but remain higher than machine learning methods, with significantly greater seasonal variation in errors. R^2^ for traditional methods decreases to 0.5–0.6 in summer and recovers only moderately in winter, indicating limited robustness under seasonal forcing. In contrast, machine learning methods show substantially reduced seasonal variability. Among them, XGB-C maintains high values R^2^ (0.85–0.95) and relatively stable error levels throughout the year, indicating temporal robustness in temperature interpolation under seasonal forcing.

Humidity interpolation exhibits stronger seasonal sensitivity and larger performance contrasts among methods due to the pronounced nonlinearity and mesoscale heterogeneity of moisture fields (Figure 8d–f). The traditional methods show peak MAE and RMSE values (purple and red curves) during summer (e.g., June–August 2023 and June–August 2024; Figure 8d,e). Although these errors decrease in winter, they remain significantly worse than those of machine learning methods. In contrast, machine learning methods (RF, XGB, XGB-C) demonstrate consistently lower errors overall. Among them, XGB-C (green curves) exhibits the smallest seasonal variability, which maintains the lowest error values throughout the period, and shows only a modest increase during summer. Although a slight reduction in R^2^ is observed during summer, XGB-C maintains higher and more stable goodness-of-fit than all other methods, demonstrating enhanced error control capability and temporal stability. In contrast, the traditional methods still underperform machine learning models, indicating insufficient robustness under varying atmospheric moisture regimes.

In surface pressure interpolation analysis (Figure 8g–i), traditional methods exhibit extremely strong seasonal sensitivity, with summer MAE and RMSE reaching approximately 8–12 hPa and 10–14 hPa, respectively. Although these metrics improve in winter, they remain an order of magnitude higher than machine learning methods. In contrast, XGB-C’s error metrics show almost no noticeable seasonal variation, maintaining extremely low levels throughout the period, with MAE and RMSE consistently around 0.15–0.35 hPa, and virtually no seasonal fluctuation, making it the most accurate and temporally stable method for pressure interpolation among the five approaches. In terms of fitting performance, the R^2^ of traditional methods drop to its lowest levels in summer (sometimes below 0), with some improvement in winter but still at relatively low levels, showing a substantial gap compared to machine learning methods. XGB-C maintains R^2^ values close to 1 throughout the year with negligible seasonal fluctuation, demonstrating far superior temporal stability in fitting compared to other methods.

In general, the interpolation effectiveness for all meteorological elements is significantly influenced by seasonal variations. During summer, increased spatial heterogeneity of meteorological elements due to factors such as complex weather systems or underlying surface conditions leads to greater interpolation challenges, manifested as increased errors (Figure 8a,b,d,e,g,h) and reduced goodness (Figure 8c,f,i) of fit, while winter performance is relatively better. Traditional interpolation methods (IDW, OK) show pronounced susceptibility to these seasonal fluctuations, with error magnitudes often increasing by 50–150% from winter to summer. Their performance is especially unstable for pressure and humidity, where summer RMSE peaks may exceed winter values by more than a factor of 2, reflecting limited robustness under dynamically evolving meteorological regimes. In contrast, machine learning methods (RF, XGB, XGB-C) display substantially smaller inter-seasonal variability and consistently higher fidelity, benefiting from their ability to learn nonlinear spatial–temporal relationships.

Quantitatively, AI-based methods achieve marked accuracy improvements relative to traditional schemes. For temperature, machine learning models reduce MAE and RMSE by approximately 40–60%, while raising R^2^ by 0.20–0.35 on average. For relative humidity, ML methods provide 50–70% reductions in MAE and RMSE and improve R^2^ by 0.25–0.40, substantially narrowing the gap in moisture field reconstruction. Pressure interpolation exhibits the most dramatic improvement: AI-driven models—especially XGB-C—lower MAE and RMSE by more than 95% compared with traditional methods, while maintaining R^2^ values near 1.0, representing an improvement of 0.70–0.90 over IDW and OK. Among all approaches, XGB-C demonstrates the greatest temporal robustness, exhibiting minimal error amplification during summer and maintaining consistently high accuracy and goodness-of-fit across the full annual cycle. With improvements of 40–70% for temperature, 60–70% for humidity, and >95% for pressure relative to traditional methods, XGB-C emerges as the most stable, accurate, and meteorologically coherent interpolation strategy among the five evaluated methods.

**Figure 9:**
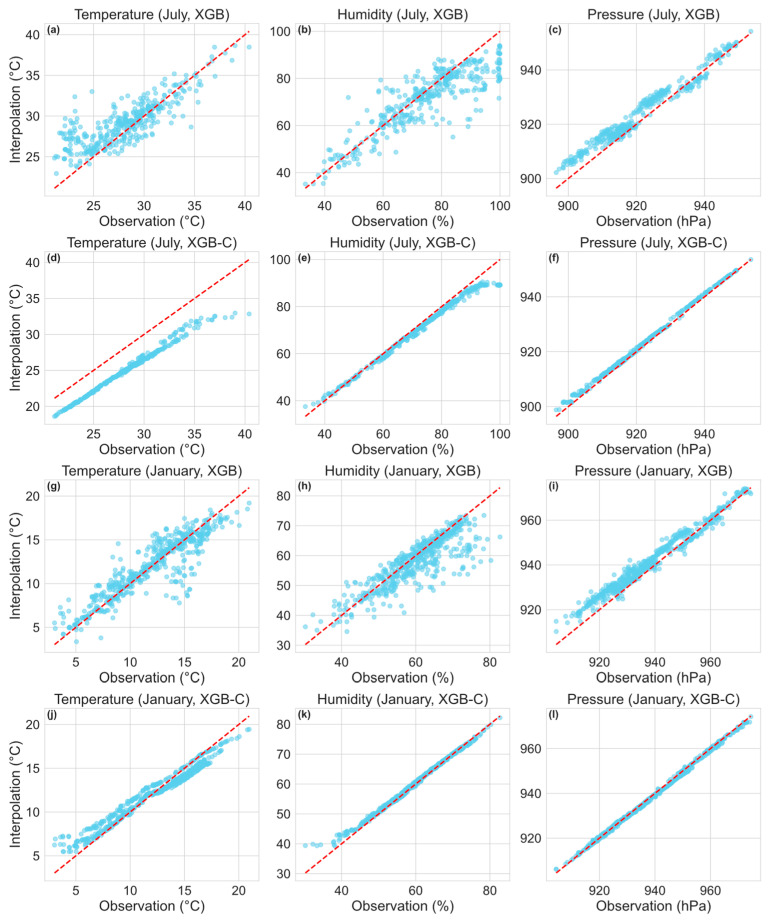
Scatter distribution of observed and interpolated temperature (°C; **left pannel**), surface pressure (hPa; **middle pannel**) and relative humidity (**right pannel**) using the (**a**–**c**,**g**–**i**) XGB and (**d**–**f**,**j**–**l**) XGB-C in winter (January) and summer (July) at test stations. In each plot, the blue dots represent the individual pairwise comparisons between observed (on the x-axis) and interpolated (on the y-axis) values, illustrating the distribution and correlation between predicted and actual data. The central trend is indicated by a red dashed line, which represents the line of equality where the interpolation perfectly matches the observation.

Given the aforementioned seasonal dependence of interpolation accuracy, even for machine learning methods, we further conducted a station-level comparison of XGB and XGB-C performance in representative winter (January) and summer (July) conditions (Figure 9). It is evident that the interpolation accuracy of XGB model exhibits stronger seasonal dependence, with scatter points showing greater deviation from the fitted line and a more dispersed distribution, indicating suboptimal fitting performance. In contrast, the XGB-C model demonstrates lower dispersion in summer temperature interpolation.

This bias may be attributed to two primary factors. First, summer is characterized by more frequent extreme weather events (e.g., rainfall, high temperatures), which may compromise sensor performance or affect station circuitry and data transmission. During data preprocessing, missing data in summer are more prevalent, resulting in incomplete feature representation for model training. Second, the XGB-C point model uses QTP15-1 as the central station (recall Figure 1), located in the river channel. Its daytime temperature is moderated by the water body and generally lower than surrounding riverbank or hillside stations, which are more directly influenced by solar radiation and surface heating. Consequently, the learned spatial mapping from the central station to other sites inherits this systematic low bias, leading to an overall underestimation in model interpolation. In summary, XGB-C exhibits superior fitting stability across winter and summer; nevertheless, extreme weather conditions in summer and the characteristics of the central station may introduce slight systematic deviations in certain variables.

### 4.3. Spatial Feature

**Figure 10:**
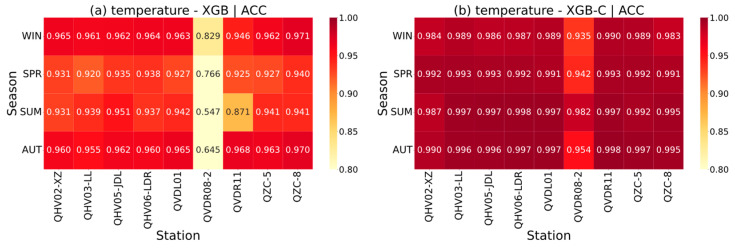
Spatial and temporal distribution of the performance of XGB-C and XGB. (WIN: winter, SPR: spring, SUM: summer, AUT: autumn).

Evaluating the spatial performance of interpolation models is essential for understanding how well they capture regional heterogeneity and reproduce physically meaningful spatial gradients in meteorological variables. Such an analysis not only reflects the model’s ability to generalize across diverse geographic environments but also helps identify systematic biases linked to the terrain, land cover, or local microclimatic effects. By examining spatial feature distributions, we can further assess whether the introduced center-station mechanism in the XGB-C model effectively enhances spatial coherence and physical consistency compared with the baseline XGB model. Figure 10 illustrates the comprehensive spatiotemporal performance of XGB-C model and XGB model. Overall, the XGB-C model demonstrates high interpolation accuracy for temperature interpolation in all the 9 test stations. In general, a widespread uniformity in interpolation performance is exhibited, though noticeable variations in meteorological states still exist among different stations (recall Figure 6), which may be primarily attributed to the proposed method’s ability to seamlessly integrate positional and geographical information. By doing so, the model effectively mitigates the typical drastic fluctuations in accuracy caused by complex topography, demonstrating high robustness across the study area. The ACC distribution of XGB-C predominantly exhibits warm tones (Figure 10a), indicating a strong correlation between interpolated and observed values and suggesting that the model effectively captures the nonlinear spatial structures of the meteorological fields. The RMSE distribution is dominated by cool tones, reflecting generally low numerical errors (Figure 10b) and highlighting the model’s robustness in controlling interpolation deviations.

From a temporal perspective, the model performance exhibits pronounced seasonal variability. In particular, interpolation accuracy in summer (July) is noticeably lower than in winter (January). This reduction in accuracy may be attributed to the increased complexity of the summer boundary-layer thermal structure, enhanced local convective and turbulent transport, and the activation of nonlinear meteorological processes, all of which increase the difficulty of fitting the temperature field. Additionally, summer is often accompanied by extreme weather events such as heavy precipitation and high temperatures. Some observation stations may experience sensor malfunction or network/electrical anomalies due to such extreme conditions, leading to partial data corruption during transmission or storage. Consequently, the higher missing data rate in summer results in less complete feature information available for model training, further challenging the model’s ability to capture local extremes.

Spatially, the XGB-C model exhibits consistently high interpolation skill across most stations, with ACC values approaching 1 and relatively small RMSE differences, indicating robust reproduction of local spatial features. In contrast, the standard XGB model shows greater spatial variability, particularly during summer 2023, reflecting its limited ability to capture fine-scale heterogeneity. During summer, the observed temperature at QVDR08-2 is lower than at the central reference station QTP15-1 (recall Figure 4a), yet the XGB-C model, which heavily relies on central-station and surrounding regional information, tends to systematically underestimate local temperatures, a phenomenon referred to as the “center-station pull effect”. Overall, these spatial patterns highlight the superior robustness of XGB-C in capturing regional meteorological variability.

In summary, the XGB-C model exhibits strong generalization capability in capturing nonlinear spatial features of meteorological fields. This enhanced performance is a direct consequence of the XGB-C modeling framework. By incorporating a center-station reference mechanism and carefully engineered spatiotemporal features, XGB-C explicitly captures the deviations of local meteorological states relative to the regional background field. This design allows the model to learn nonlinear spatial gradients and local heterogeneity more effectively than standard XGB, ensuring that spatial interpolation reflects physically plausible patterns such as vertical temperature lapse, pressure decay with altitude, and humidity variability constrained by terrain. However, its ability to accurately fit localized micro-scale topographic effects, seasonal nonlinear processes, and data gaps remains limited. Enhancing local interpolation accuracy may require incorporating terrain-derived variables, locally weighted features, or residual correction strategies.

## 5. Conclusions

Generating high-resolution meteorological fields for atmospheric refraction correction in mountainous hydropower is a long-standing challenge. In this study, three machine learning approaches and two traditional methods for high-precision interpolation of meteorological fields around the Xiluodu Hydropower Station are designed and compared. Using daily data from 47 stations (38 training, 9 testing) over 2023–2024, we compared IDW and Ordinary Kriging with Random Forest, XGBoost, and a center-point-enhanced XGBoost (XGB-C). Machine learning consistently outperforms traditional methods across variables and seasons, with XGB-C achieving the highest accuracy (R^2^ ≈ 1.00 for surface pressure, 0.97 for relative humidity, 0.83 for temperature) and the lowest MAE/RMSE, indicating superior capacity to capture nonlinear spatial variability.

Performance exhibited clear temporal variation. All methods degraded in summer, but the seasonal sensitivity was much stronger for IDW/OK than for ML models. For XGB-C, accuracy declines only modestly in July relative to January, where R^2^ remains high, reflecting better temporal stability, which may be attributed to the higher capacity in representing the nonlinear features of atmospheric states. The summer drop is attributable to more complex boundary-layer thermodynamics, enhanced local convection/turbulence, and higher rates of missing or corrupted observations during extremes, which reduce feature completeness and increase the difficulty of resolving local extremes, particularly for temperature. In addition, spatial heterogeneity was evident. Stations in “Urban and Built-Up” and “Croplands” land-use classes show larger errors, and a notable “central-station pull effect” emerged at QVDR08-2, where the model systematically underestimates local temperature relative to the river-channel anchor station (QTP15-1). This bias suggests that models relying primarily on inter-station spatial features may smooth microclimatic anomalies and under-represent localized cold- or heat-island effects in areas with sharp gradients and limited representativeness.

This study demonstrates that the operational value of machine learning—especially tree-ensemble methods—for precise interpolation of temperature, humidity, and pressure in rugged terrain directly relevant to geodetic correction. Second, the proposed center-point XGBoost (XGB-C) introduces a simple but effective feature-anchoring strategy that encodes relative spatial differences to the central station, yielding consistent gains over both standard XGB and RF and delivering near-constant accuracy for pressure. Given that the terrain near the Xiluodu Dam is highly complex and our method was successfully applied there, we are confident that it can be readily generalized to most regions with simpler terrain, we have added this discussion in the revised manuscript. However, limitations exist due to insufficient observations. Future work is warranted to augment input features with terrain-derived variables (elevation derivatives, aspect, distance-to-water), land-surface and boundary-layer proxies (radiation, wind, stability), and physics-informed constraints (lapse rates, hydrostatic relationships). Residual correction via regression- or universal-kriging hybrids, locally weighted models, and spatial cross-validation that accounts for autocorrelation would likely reduce local biases and over-smoothing. Robust handling of missing and extreme-event data, uncertainty quantification, and tests across longer periods and additional stations or remote sensing covariates (e.g., DEM, LST) are also needed to strengthen generalizability and operational readiness. In the next work, we will focus on developing fully operational models by training them with similar ML techniques. This will be achieved by leveraging extensive, high-quality labels derived or retrieved from multi-source remote sensing data, including high-resolution satellite imagery and observation-based unmanned aerial vehicles.

## Figures and Tables

**Table 1:** Geographic information of the test stations.

Station	B (°)	L (°)	H (m)	Slope (°)	Location	Land-Use
QHV02-XZ	28.254863	103.6577	472.4742	45.2220993	South-West	Urban and Built-Up
QHV03-LL	28.263238	103.65224	778.3022	26.60479927	South	Croplands
QHV05-JDL	28.264053	103.6515	815.1362	14.90699959	South-West	Croplands
QHV06-LDR	28.254961	103.64878	771.5702	29.3302002	North-East	Evergreen Broadleaf Forest
QVDL01	28.264114	103.64936	754.8342	43.51190186	South-West	Woody Savannas
QVDR11	28.25764	103.63161	892.2922	41.42610168	North-West	Evergreen Broadleaf Forest
QZC-5	28.257806	103.65622	665.7572	40.53480148	South-West	Croplands
QZC-8	28.25205	103.6545	660.7572	35.17499924	North-East	Croplands
QVDR08-2	28.259575	103.65363	561.416	52.05979919	North-East	Urban and Built-Up

**Figure A1:**
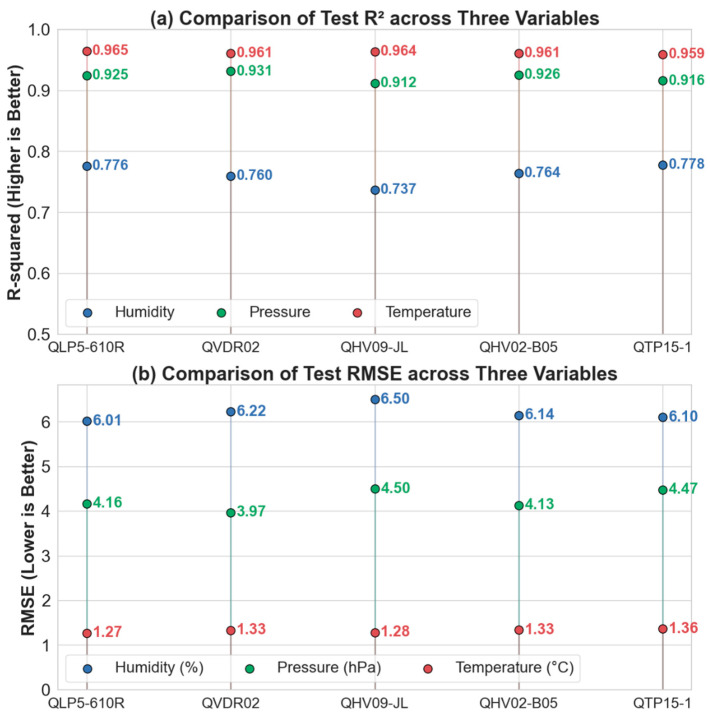
Comparison of interpolation accuracy across the top representative central stations for three meteorological variables.

**Figure A2:**
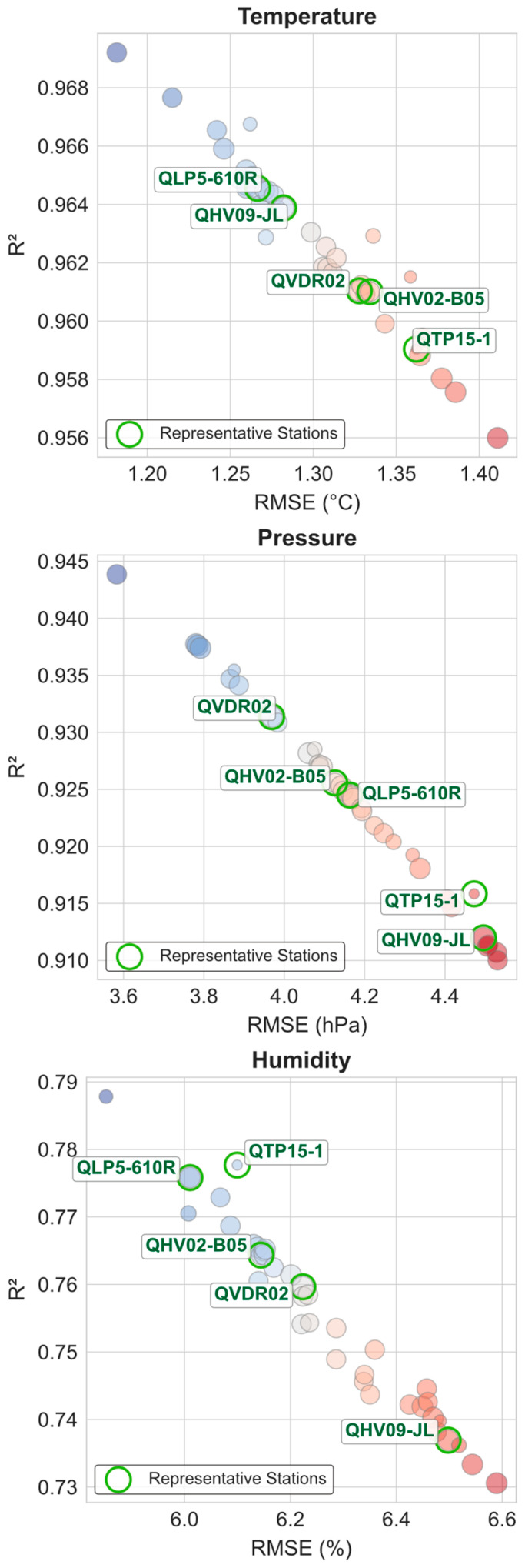
Performance distribution (RMSE vs. R^2^) of the XGB-C model for all variables (temperature, humidity and pressure) across all candidate central stations. Circles with green borders denote the stations identified by the representativeness framework. For visual clarity, only the models associated with the top 35 ranked stations are displayed, with text labels highlighting the top five representative stations. The color of each circle represents the relative performance ranking of the stations, with cooler colors (blue) indicating better performance (lower RMSE and higher R^2^), and warmer colors (red) indicating poorer performance (higher RMSE and lower R^2^). The green ring markers highlight the selected representative stations.

## Data Availability

The ERA5 data are available at https://cds.climate.copernicus.eu/datasets (accessed on 14 March 2026).

## Abbreviations

The following abbreviations are used in this manuscript:

AI	Artificial Intelligence
ACC	Accuracy Correlation Coefficient
NCEP	National Centers for Environmental Prediction
CMONOC	Crustal Movement Observation Network of China
ML	Machine Learning
IDW	Inverse Distance Weighting
OK	Ordinary Kriging
XGB	eXtreme Gradient Boosting
RF	Random Forest
UAVs	unmanned aerial vehicles
SVR	Support Vector Regression
RMSE	Root Mean Square Error
MAE	Mean absolute error
R^2^	Coefficient of Determination

## Appendix A

To evaluate the sensitivity of the XGB-C model to the selection of the central station, we have conducted an exhaustive experiment by rotationally setting each training station as the central station. Beyond model performance alone, the selection of the central station should also have geographical and physical representativeness of the regional meteorological background. Therefore, we established a Station Representativeness evaluation framework to quantitatively assess how well each station reflects the regional meteorological background. The framework integrates three complementary criteria derived from both statistical and geographical perspectives. First, temporal evolution consistency was evaluated by calculating the mean Pearson correlation coefficient between the time series of a candidate station and those of all other N−1 stations. This metric quantifies the station’s ability to capture the dominant temporal variability of the regional meteorological field. The temporal evolution consistency (${\bar{R}}_{c}$) was calculated as

$${\bar{R}}_{c}=\frac{1}{N-1}\sum_{i=1,i\neq c}^{N}Cor(S_{c},S_{i})$$

where the N denotes the total number of stations, $S_{c}$ denotes the time series of candidate central station, $S_{i}$ denotes the time series of other stations i, Cor(.) denotes Pearson correlation coefficient. A ${\bar{R}}_{c}$ value closer to 1 indicates that the station’s fluctuations are highly synchronized with the regional trend.

Second, magnitude proximity was quantified by the root mean square error (RMSE) between the station’s time series observation and the regional mean time series, which was obtained by averaging observations from all stations at each time step. The RMSE were calculated as

$$RMSE_{c}=\sqrt{\frac{1}{T}\sum_{t=1}^{T}(S_{c}(t)-\bar{S}(t){)}^{2}}$$

where the T is the total number of time steps and $\bar{S}(t)$ denotes regional mean at time t. A smaller $RMSE_{c}$ indicates that the station more closely represents the regional average magnitude of the meteorological variable.

Third, geo-topographical centrality was assessed based on the spatial and elevation offsets relative to the regional geometric center of the observation station network. The regional geometric center ($\bar{x},\bar{y},\bar{h}$) was defined by the mean horizontal coordinates and mean elevation of all stations. For a given station i with coordinates ($x_{i}$, $y_{i}$) and elevation $h_{i}$, the horizontal spatial deviation $D_{i}$ and elevation deviation $E_{i}$ were calculated as:

$$D_{i}=\sqrt{(x_{i}-\bar{x}{)}^{2}+(y_{i}-\bar{y}{)}^{2}}$$

$$E_{i}=|h_{i}-\bar{h}|$$

Stations with smaller spatial and elevation deviations are considered more geographically representative of the regional meteorological environment.

These geographical indicators describe whether a station occupies a physically central position within the observation network and the regional terrain structure. Stations were subsequently ranked based on their overall representativeness, giving priority to stations that exhibited lower RMSE relative to the regional mean, higher mean correlation with other stations, and smaller spatial and elevation deviations. This combined ranking enables an objective selection of the most representative central station for the XGB-C model. Stations that are ranked within the top 10 across all evaluated representativeness metrics for temperature, humidity, and pressure: QTP15-1, QLP5-610R, QHV09-JL, QHV02-B05, QVDR02.

As illustrated in Figure A1, the XGB-C model exhibits remarkable robustness across different candidate central stations. The performance metrics (R2 and RMSE) remain highly stable among the top ten representative stations, particularly for temperature and pressure, where the R2 values consistently exceed 0.95 and 0.91, respectively. Although humidity shows a slightly higher sensitivity to station selection, with RMSE fluctuating between 6.01 and 6.50, the overall variance remains within a negligible range (<0.05 for R2). This consistency validates that the representative power of the selected central station is sufficiently high to ensure the stability of the regional interpolation framework, further justifying our choice of the dam-site station as the primary central node for practical engineering applications.

As illustrated in the composite scatter plots (Figure A2), the XGB-C model demonstrates high stability across different central station configurations for all three meteorological variables. A strong inverse linear relationship between RMSE and R2 is observed across all candidate models, confirming the reliability of the performance metrics. Notably, while the absolute highest R2 values are achieved by a small subset of stations, the “representative stations” identified by our framework (e.g., QTP15-1) consistently maintain high-tier performance. For instance, in temperature modeling, the R2 variance among the top 35 stations is less than 0.015. This minimal sensitivity justifies the prioritization of stations with high geo-topographical centrality and engineering relevance, ensuring that the spatial interpolation framework is both accurate and physically representative of the regional meteorological background.

The results indicate that while the current station (QTP15-1) used in our study is not the absolute best-performing candidate among all stations, the differences in performance among highly representative stations are minimal. This suggests that the XGB-C framework is relatively insensitive to the specific choice of the central station when the selected station adequately represents the regional meteorological background. In addition to statistical performance, practical considerations related to the engineering environment of the Xiluodu Hydropower Station were also taken into account. The station used as the central node in this study is located at the dam site, which occupies a geographically strategic position near the center of the study area. This location effectively balances the distinct microclimatic influences of the north and south banks, which differ in slope orientation and terrain characteristics. As a result, the dam-site station serves as a stable reference point for capturing the background meteorological conditions of the region and plays an important role in operational monitoring at the hydropower facility.

It should also be noted that in this sensitivity experiment the hyperparameters of the XGB-C models were not independently optimized for each central-station configuration. Consequently, the overall accuracy reported in this appendix may differ slightly from the optimized results presented in the main manuscript. Nevertheless, the consistent behavior observed across the sensitivity experiments confirms that the central station selected in this study provides a representative and robust reference for the XGB-C modeling framework within the study area.
